# Oral microbiota in cardiovascular health and disease

**DOI:** 10.3389/fcimb.2025.1731845

**Published:** 2026-01-02

**Authors:** Yi-Chuan Wu, Bo-Yan Chen, Sheng-Zhong Duan

**Affiliations:** Stomatology Hospital, School of Stomatology, Zhejiang University School of Medicine, Zhejiang Provincial Clinical Research Center for Oral Diseases, Key Laboratory of Oral Biomedical Research of Zhejiang Province, Cancer Center of Zhejiang University, Engineering Research Center of Oral Biomaterials and Devices of Zhejiang Province, Hangzhou, China

**Keywords:** cardiovascular disease, inflammation, oral microbiome, oral-heart axis, periodontal health

## Abstract

Hundreds of microbial species inhabit the oral cavity and are essential to local and systemic health. Cardiovascular disease (CVD) causes a significant burden on global health, with its etiology and pathogenesis still not fully understood. Growing evidence indicates an association between the oral microbiome and CVD. However, the variability of populations, follow-up durations, and disease severity between various human investigations led to divergent conclusions. Animal studies have focused on the relationship between individual microbial species and CVD, yet gaps remain in the mechanisms between CVD and microbiome. In addition, a comprehensive understanding of the interactions between oral pathogens and the cardiovascular system has not been elucidated. In this Review, we summarize our current knowledge of basic and clinical research on potential mechanisms linking the oral microbiome to CVD and highlight the need for population-based studies with controlled baselines to establish mechanism links between oral microbial pathways and CVD.

## Introduction

The oral cavity is the second-largest human microbial habitat, colonized by more than 700 microbial species, which can be mainly divided into *Actinomycetota, Bacteroidetes, Firmicutes, Fusobacteria, Proteobacteria, Saccharibacteria, and Spirochaetes (*Baker et al., 2024; Chandra Nayak et al., 2025). Approximately 300 species-level taxa constitute an oral microbial profile across a typical individual, and they are generally conserved (Kilian et al., 2016). However, different parts of the oral cavity harbor a microbiome with unique characteristics and functional roles. For instance, the supragingival microbiome is commonly associated with dental caries, whereas subgingival plaques are more closely linked to the development of periodontal disease and oral squamous cell carcinoma (Lamont et al., 2018; Narayanan et al., 2023). With the cost reduction of high-throughput sequencing and technological advancements, the relationship between human health and the oral microbiome has been gradually revealed, with more than oral bacteria. Viruses, fungi, and archaea within the oral cavity are associated with various diseases (Stasiewicz and Karpiński, 2022; Teles et al., 2022). Moreover, accumulating evidence shows the link between the oral microbiome and systemic diseases, including inflammatory bowel disease, colorectal cancer, Alzheimer’s disease, and rheumatoid arthritis, of which cardiovascular disease has attracted much attention (Baker et al., 2024). Consequently, understanding the interaction between the host and their oral microbiome is pivotal for elucidating the cause of cardiovascular disease (CVD) and designing targeted therapeutic interventions.

The global prevalence of CVD has increased markedly over the past thirty years, thereby exerting a substantial socioeconomic burden and emerging as the foremost cause of mortality worldwide (Libby et al., 2019). Emerging studies have demonstrated the multiple effects of the human microbiome on modulating CVD in various ways, including metabolites, host immune-inflammatory responses, and bacteremia (Szegedi et al., 2025). Various reports indicate that oral infections caused by pathogens, especially periodontitis, are the most common disease in humans and a risk factor for CVD. Periodontal pathogens have been found to exacerbate atherosclerosis through causing bacteremia (Priyamvara et al., 2020). Moreover, elevated systemic inflammation and periodontitis were linked to cardiovascular mortality (Plachokova et al., 2021). Oral hygiene care, such as frequent brushing and periodontitis treatment, not only led to improved vascular endothelial function, but also favorable changes in carotid intima-media thickness and blood pressure, and ultimately reduces the risk of cardiovascular events (Park et al., 2019; Schiffrin and Engert, 2019; Czesnikiewicz-Guzik et al., 2020). There is consistent and relatively strong epidemiologic evidence linking the impact of the oral microbiome on cardiovascular health and further suggesting the potential role of oral hygiene care in improving cardiovascular outcomes. However, the causal association and pathophysiological mechanisms of the effect of the oral microbiome on CVD are complex and not fully understood. Recent researches implicate that immune system changes provoked by oral pathobionts may be partially responsible for aggravated CVD. It has been demonstrated that a local periodontal inflammation can drive the activation of remote inflammatory B lymphocytes and neutrophils in infarcted mouse hearts (Chen, 2025; Wang et al., 2025). Immunization of hypertensive mice using *Porphyromonas gingivalis* (*P. gingivalis*) and *Filifactor alocis* deteriorates hypertension via increased susceptibility to raised blood pressure with infiltration of interferon-γ^+^ T cells (Czesnikiewicz-Guzik et al., 2019; Zhang et al., 2025). In summary, a growing body of evidence from epidemiological studies, basic science, and clinical investigations supports the profound effect of the oral microbiome on CVD, and a comprehensive discussion of current studies could improve our understanding of the relationship between CVD risk and oral microbiome.

Here, we characterized the relationship between oral microbiome and CVD, including hypertension, atherosclerosis, myocardial infarction, endocarditis, and heart failure, aiming to identify that mouth-cardiovascular system correlations are not only a consequence of common risk factors but are also driven to some extent. We also discussed the underlying mechanisms of the oral microbiome in the progression of CVD. Overall, this Review provides perspectives on the known relevance and mechanisms of CVD progression related to the oral microbiome and offers a foundation for future disease prevention and treatment strategies.

## Oral microbiome and hypertension

Hypertension is a significant health issue affecting more than 1.2 billion people worldwide, which is closely correlated with the oral microbiome (Zhou et al., 2021). Higher oral microbial diversity was significantly associated with a lower all-cause mortality risk in American hypertensive individuals (Yang et al., 2024). Elevated abundance of several salivary bacteria, including *Atopobium, Prevotella*, *Bacteroides*, *Streptococcus*, and *Veillonella*, was found in hypertension patients (Marchi-Alves et al., 2017; Murugesan and Al Khodor, 2023; Al-Maweri et al., 2025). Imbalance of oral microbiota is an important pathogenic factor for periodontitis, and its links with hypertension have been extensively studied. Higher burden of etiologic bacteria and antibodies of periodontitis was significantly associated with higher SBP, DBP, and hypertension prevalence (Desvarieux et al., 2010; Pietropaoli et al., 2019). Various periodontal bacteria, including *Prevotella intermedia* (*P. intermedia*), *P. gingivalis*, and *Fusobacterium nucleatum* (*F. nucleatum*), have been reported to be significantly associated with hypertension (da Silveira et al., 2022; Leonov et al., 2024). Oral viromes such as *Gillianvirus* were associated with lower hypertension risk, and mycobiome *Torbevirus* and *Exophiala* sp*inifera* were positively related to hypertension (Chen et al., 2023b; Ye et al., 2024). Antihypertensive medications may alleviate inflammation and bone loss in mice with periodontitis, while antihypertensive treatment aggravated oral microbiome dysbiosis in periodontitis patients (Li et al., 2019; Kim et al., 2023). The possible reasons for the varied results may be due to different medication types and timing.

**Figure 1:**
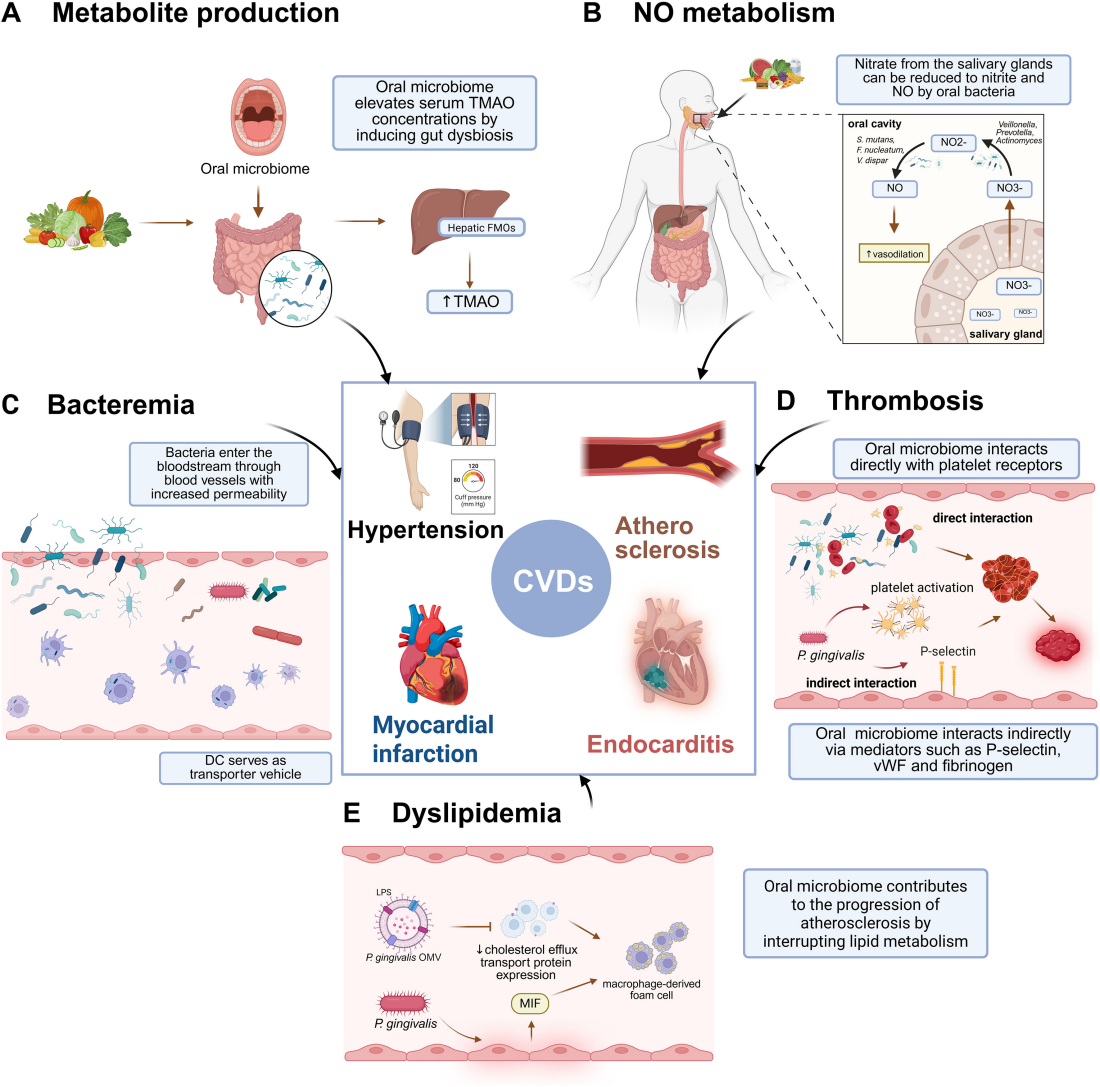
Oral microbiome in the pathogenesis of cardiovascular diseases. **(A)** Elevated microbial metabolites (TMAO) caused by periodontitis induced gut dysbiosis and impaired endothelial function, promoting the development of CVD. **(B)** Oral microbiome affects hypertension through the regulation of NO levels. **(C)** Bacteremia caused by periodontal pathogens directly elicits endothelial dysfunction or induces inflammation by invading immune cells, contributing to CVD. **(D)** Platelet aggregation evoked by oral species assumes a crucial role in advancing atherosclerosis. **(E)** The alteration of lipid metabolism is an essential mechanism for the influence of the oral microbiome on atherosclerosis. The figure was Created in BioRender (https://BioRender.com/hxdyk8u).

Oral microbiota translocation is involved in the progression of hypertension. Although most microorganisms are killed by the stomach and bile acids under physiological conditions, oral microbes can directly enter the gut through swallowing, especially in the periodontal infection condition (Yamamoto et al., 2023). *Porphyromonadaceae* and *Fusobacterium* from severe periodontitis patients could translocate and colonize in the mouse gut (Bao et al., 2022). *P. gingivalis* entered human aortic endothelial cells by invading DC as transporter vehicles through FimA fimbriae, posing a threat to hypertension (Hajishengallis, 2015). *Veillonella* was a frequent oral-gut transmitter stably enriched in HTN patients and hypertensive mice receiving human oral microbiota transplantation (Chen et al., 2023c). Moreover, the oral microbiome contributes to the regulation of hypertension through modulation of gut-derived metabolites. Elevated trimethylamine N-oxide (TMAO) was found in the serum of severe periodontitis patients and ApoE^-/-^ mice with experimental periodontitis, causing gut dysbiosis and impaired endothelial function (**Figure 1A**) (Xiao et al., 2021; Zhou et al., 2022). Distinct metabolic clusters in hypertensive patients were significantly correlated with microbiome in subgingival plaque (Chen et al., 2023a).

Periodontal pathogens aggravate hypertension by inducing endothelial dysfunction. An increased flow-mediated dilation (FMD) of the brachial artery was found among patients with periodontitis (Holtfreter et al., 2013). Various studies have also recommended periodontal treatment to improve endothelial function and decrease potential biomarkers (Li et al., 2022). Several pathways of periodontitis have been identified as contributing to the progression of endothelial dysfunction. Bacteremia caused by periodontal pathogens may elicit endothelial inflammation (Hajishengallis, 2015). Meanwhile, the pro-inflammatory mediators from severely infected periodontal tissue can be released into the systemic circulation, which in turn affects endothelial function. The host immune system responds to microbial infection, thus varying vascular endothelial injury among host individuals with different susceptibilities (Li et al., 2022). Periodontal inflammation can be detected by receptors on vascular endothelial cells, including toll-like receptor-2 (TLR-2) and TLR-4, thus activating the inflammatory cascade, which releases pro-inflammatory cytokines and promotes the expression of adhesion molecules (Schenkein and Loos, 2013; Chen et al., 2021).

Oral microbiome affects hypertension through the regulation of nitric oxide (NO) levels (**Figure 1B**). NO from endothelial cells has been found to act as a vasodilator. Endogenous NO is produced by specific NO synthases (NOS) using oxygen and NADPH to oxidize L-arginine to L-citrulline (Pignatelli et al., 2020). NOS can be mainly classified into three categories: neuronal NOS, inducible NOS, and endothelial NOS. The eNOS has been considered the regulator of endothelial integrity and vascular tone (Luo et al., 2018; Rajapakse et al., 2019). The released NO can be immediately oxidized to nitrite and nitrate, and the nitrate from the salivary glands and diet can be reduced to nitrite by nitrate reductase enzymes in oral bacteria, which subsequently reduces to NO by denitrifying bacteria in the oral cavity^54–57^. A case-control study found higher salivary NO and microbial diversity in normotensive individuals (Barbadoro et al., 2021). Moreover, an increased dietary nitrate intake from a vegetable source has also been shown to modulate the oral microbiome and promote an increased abundance of nitrate-reducing species (Fejes et al., 2025). Various oral microbiomes, including the genera *Neisseria*, *Haemophilus*, *Granulicatella*, *Veillonella*, *Prevotella*, *Corynebacterium*, *Actinomyces*, and *Rothia*, have been found to reduce nitrate. *Streptococcus mutans, F. nucleatum*, and *Veillonella dispar* were identified as capable of nitrite reduction (Morou-Bermúdez et al., 2022). Meanwhile, *P. gingivalis*-infected endothelial cells possessed excessive ROS production, which may interfere with the NO pathway and reduce NO bioactivity (Garcia and Sessa, 2019; Xie et al., 2021). However, NO release also has antimicrobial activity against periodontitis-associated species, including *Aggregatibacter actinomycetemcomitans* (*A. actinomycetemcomitans*), and *F. nucleatum (*Rosier et al., 2022). Antibacterial mouthwash induces compositional changes in the oral microbiome, potentially compromising NO production. Several studies have shown that using an antibacterial mouth rinse decreased the concentrations of salivary and plasma nitrate/nitrite, leading to a concomitant rise in blood pressure (Bondonno et al., 2015; Senkus and Crowe-White, 2020). A mouth rinse infused with inorganic nitrate and antioxidants can influence the diversity and construction of the oral microbiome, promoting entero-salivary circulation and contributing to a reduction in blood pressure within one week (Senkus et al., 2025). However, another meta-analysis did not show a significant relationship between mouth rinse use and blood pressure (Farook et al., 2024). The possible reasons for the inconsistency may include varied composition and duration of mouthwash use and different severity of hypertension. The released NO can be immediately oxidized to nitrite and nitrate, and the nitrate from the salivary glands and diet can be reduced to nitrite by nitrate reductase enzymes in oral bacteria, which subsequently reduces to NO by denitrifying bacteria in the oral cavity (Lundberg et al., 2008).

## Oral microbiome and atherosclerosis

The empirical pathological features of atherosclerosis are the accumulation of fatty and/or fibrous material in the innermost layer of arteries, the intima (Libby et al., 2019). Chronic inflammation triggers macrophage infiltration and cholesterol accumulation in the vessel wall. The immune cascade contributes to the proliferation of smooth muscle cells, the accumulation of connective tissue components, and the oxidation of low-density lipoprotein (LDL), which subsequently leads to plaque rupture and thrombus formation, ultimately resulting in tissue ischemia and necrosis (Weber and Noels, 2011). Systemic inflammation elicited by the oral microbiome may act as a mediator between chronic diseases, including atherosclerosis and periodontitis. Periodontitis patients showed an elevated leukocyte count, TNF-α, IL-8, IL-6, and active MMP-8 (aMMP-8) level, posing a threat to endothelial dysfunction and contributing to the incidence of atherosclerosis (Pink et al., 2015; Noack et al., 2017; Ribeiro et al., 2021). The oral microbiome is also directly linked to atherosclerosis. Patients with atherosclerosis have a high prevalence of *Streptococcus, Lactobacillus, Actinobacteria*, and *Cutibacterium*, and a low abundance of *Bacteroidetes*, in the saliva of the oral cavity (Ueno et al., 2012; Bouzid et al., 2022; Kato-Kogoe et al., 2022; Nazari et al., 2024; Wu et al., 2024). *Veillonella* and *Streptococcus* of dental plaque have been detected in both dental and atherosclerotic plaques (Koren et al., 2011). Specifically, for symptomatic atherosclerosis, increased oral *Anaeroglobus* abundance was identified (Fåk et al., 2015). Cariogenic and periodontal species have been identified in atherosclerotic plaques (Sato et al., 2024). The DNA of *A. actinomycetemcomitans, Pseudomonas* sp*ecies, P. gingivalis, S. mutans, Acinetobacter, Tannerella forsythensis*, and *Viridans streptococci* have been found in the atherosclerotic plaques (Nakano et al., 2006; Nakano et al., 2007; Armingohar et al., 2014; Calandrini et al., 2014; Fernandes et al., 2014; Mougeot et al., 2017; Kannosh et al., 2018; Rao et al., 2023). At the same time, viable oral bacteria have not been isolated from the atheromas (Fiehn et al., 2005). Although inducing transient bacteremia, periodontal therapy has been found to reduce systemic inflammation, suggesting its protective role for atherosclerosis among periodontitis-affected patients (Waghmare et al., 2013; Herrera et al., 2020).

Oral microbiome fuels atherosclerosis development via multiple mechanisms. Bacteremia can arise from periodontal therapy, daily activities like tooth brushing and chewing, and bacteria invading circulating immune cells (**Figure 1C**) (Herrera et al., 2020). Moreover, *P. gingivalis* can induce the phenotype transformation of smooth muscle cells, resulting in aortic intimal hyperplasia (Hokamura et al., 2010). Notably, specific oral species, including *P. gingivalis, F. nucleatum, A. actinomycetemcomitans, P. intermedia*, and *T. denticola*, significantly promote the progression of atherosclerosis. The pathogenic mechanisms of *P. gingivalis* on atherosclerosis are the most extensively studied. *P. gingivalis* LPS triggers inflammatory responses by binding to TLR2 or TLR4 receptors, activating NF-κB transcription, which may cause endothelial dysfunction and enhance atherosclerosis (Aleksijević et al., 2022). Gingipains from *P. gingivalis* increase capillary permeability via the peptide bond-hydrolyzing activity, degrade α- and β-defensins, reduce CD14 receptor expression on macrophages, and break down complement components C3, C4, and C5, which contribute to inflammation and promote the development of atherosclerosis (Aleksijević et al., 2022). *P. gingivalis* fimbriae are classified into major FimA and minor Mfa forms, and the former one could adhere to hydroxyapatite and oral epithelium (Sojar and Genco, 2005). Fimbriae also mediate *P. gingivalis* entry into dendritic cells, aiding bacteremia, subsequently resulting in atherosclerosis (El-Awady et al., 2022). The FimA, Mfa, and small RNA in *P. gingivalis* outer membrane vesicles (OMVs) facilitate cell communication, and these OMVs are present during different stages of atherogenesis (Zhang et al., 2021). *P. gingivalis* OMV can compromise endothelial integrity by disrupting junction proteins and activate the ERK1/2 signaling pathway, increasing the expression of the osteogenic transcription factor Runx2 in vascular smooth muscle cells, promoting their differentiation into osteoblast-like cells, enhancing mineralization, and contributing to vascular calcification (Lin et al., 2025). *F. nucleatum* promotes atherosclerosis through several mechanisms. FadA enhances endothelial permeability, while heat-shock protein GroEL upregulates chemokines and tissue factor expression, facilitating monocyte adhesion and transmigration (Nithianantham et al., 2006; Fardini et al., 2011; Fan et al., 2023). Interestingly, a beneficial effect was observed in mice infected solely with *F. nucleatum (*Velsko et al., 2015). Further research is needed to understand the protective mechanisms, the role of virulence factors, and interactions with other pathogens in atherosclerosis progression. Leukotoxin LtxA *of A. actinomycetemcomitans* helps evade immunity by binding to β2 integrin LFA-1 on leukocytes (Vega et al., 2019). Additionally, *P. intermedia’*s lipase and *T. denticola*’s chymotrypsin-like protease (Td-CTLP) contribute to tissue degradation and promote atherosclerosis (Lund Håheim, 2024; Wang et al., 2024).

Platelet aggregation evoked by oral species assumes a crucial role in advancing atherosclerosis (**Figure 1D**). Increased platelet activation has been observed in patients with generalized aggressive periodontitis (Zhan et al., 2016). Furthermore, elevated levels of PAI-1 have been identified in the plasma of individuals with periodontitis (Bizzarro et al., 2007). The oral microbiome can interact directly with platelet receptors or indirectly via mediators such as von Willebrand factor (vWF) and fibrinogen (Gualtero et al., 2023). *P. gingivalis* has been associated with the activation of platelets through the binding of RgpA and RgpB to hemagglutinin/adhesion molecules, which subsequently stimulate Par-1 and Par-2 (Lourbakos et al., 2001). *P. gingivalis* promotes platelet activation by increasing P-selectin expression and facilitating fibrinogen binding to platelets (Yu et al., 2011).

Metabolic changes are the essential mechanisms for the influence of the oral microbiome on atherosclerosis (**Figure 1E**). *F. nucleatum* contributes to the progression of atherosclerosis by stimulating hepatic lipogenesis (Zhou et al., 2023). *P. gingivalis* OMV carrying LPS has been demonstrated to inhibit the transcription of cholesterol efflux transport proteins, thereby facilitating the formation of macrophage-derived foam cells (Lin et al., 2025). Furthermore, *P. gingivalis* can promote foam cell formation by upregulating the expression of macrophage migration inhibitory factors in endothelial cells (Chen et al., 2017; Xu et al., 2018). Elevated levels of TMAO have been associated with an increased risk of cardiovascular diseases, as they influence inflammatory processes, cholesterol metabolism, and thrombosis (Warrier et al., 2015; Zhu et al., 2016; Chen et al., 2017). Trimethylamine (TMA), generated through the microbial degradation of nutrients such as carnitine, choline, and lecithins within the gut, undergoes oxidation to form TMAO via the hepatic enzyme flavin monooxygenase (FMO) -3 (Bennett et al., 2013). The oral microbiome can elevate serum TMAO concentrations by inducing gut dysbiosis, which can be ameliorated through periodontal therapy (Gan et al., 2023; Gan et al., 2024; Huang et al., 2025).

Numerous studies have concentrated on the role of innate immune cells in periodontitis and inflammatory comorbidities. Elevated levels of neutrophils, monocytes, and lymphocytes have been detected both in periodontitis and atherosclerosis (Wu et al., 2024). Trained immunity, defined as the enhanced state of immune response of innate immune cells induced by stimulation, enabling them to respond more effectively to subsequent challenges, has been identified as one of the mechanisms contributing to both periodontitis and atherosclerosis through the promotion of systemic inflammation (Li et al., 2023; Ochando et al., 2023; Riksen et al., 2023; Wu et al., 2024). As a widely studied immune adjuvant, cyclic diadenylate monophosphate (c-di-AMP) has been shown to mitigate the worsening effects of P. gingivalis on atherosclerosis by activating trained immunity (Wu et al., 2023). Clonal hematopoiesis of indeterminate potential (CHIP) represents another mechanism that links periodontitis with inflammatory comorbidities, including atherosclerosis (Jaiswal and Libby, 2020; Mitroulis et al., 2024; Wang et al., 2024). Nonetheless, the specific role of innate immune cells in periodontitis and atherosclerosis remains insufficiently investigated and warrants further exploration in future studies.

## Oral microbiome and myocardial infarction

The association between myocardial infarction (MI) and oral microbiome has aroused widespread concern (Mattila et al., 1989). Distinct alterations in oral and gut microbiome composition occurred in MI patients (Khan et al., 2025a; Khan et al., 2025b; Lei et al., 2025). More severe periodontitis has been found in MI patients, with higher probing depths and abundance of *P. gingivalis* and *Prevotella* genus (Stein et al., 2009; Pasupuleti et al., 2013; Seoane et al., 2022; Hernández-Ruiz et al., 2023). Low levels of antibodies to *T. forsythia* can predict the cardiovascular disease mortality of MI patients (Lund Håheim et al., 2020). Moreover, oral *Viridans Streptococci* and *A. actinomycetemcomitans* have been detected in the plaque of MI patients (Pessi et al., 2013; Calandrini et al., 2014). *Streptococcus* spp. can transmit from the oral to the gut, thus exacerbating MI in mice (Li et al., 2025). *P. gingivalis* invasion was detected in the ischemic myocardium, and its LPS could activate CD8^+^ T cells, apoptosis, and the MMP-9 activity, causing cardiac rupture (DeLeon-Pennell et al., 2014; Shiheido et al., 2016; Zaidi et al., 2021). *P. gingivalis* gingipains could also impair the infarcted myocardium via cleaving VAMP8 and interrupting autophagy (Shiheido-Watanabe et al., 2023). Elevated IL-17A was also detected in MI mice with experimental bacteremia of *P. gingivalis (*Akamatsu et al., 2011). Recently, oral microbiomes and the proinflammatory B2 cells have been shown to contribute to the progression of MI (Chen, 2025).

Moreover, TMAO concentration in the plasma was positively associated with the abundance of *P. gingivalis* among MI patients (Hernández-Ruiz et al., 2024). Circulating short-chain fatty acids (SCFA), including isobutyric and 2-methylbutyric acids, were significantly elevated in MI patients and correlated with oral and gut microbiome (Constantino-Jonapa et al., 2025). Specifically, butyrate has been found to promote cardiac repair through the recruitment and activation of monocytes and neutrophils to the peri-infarct region (Jiang et al., 2020). Certain oral bacteria produce SCFA in periodontal pockets that can contribute to both beneficial and harmful effects in the oral cavity (Guan et al., 2021; Leonov et al., 2023). Propionate and butyrate promoted T cell polarization and the development of extrathymic regulatory T cells, followed by their activation in rodent models of MI, thereby mitigating inflammatory damage in the myocardium (Bijla et al., 2024). However, the specific oral species mediating SCFA production and the underlying mechanisms remain to be explored.

## Oral microbiome and endocarditis

Infective endocarditis (IE) is a disease that involves infection of the heart valves, with an increasing cases and mortality in recent years (Li et al., 2024). *Staphylococcus* is the primary pathogen causing IE, while Streptococcus is the most commonly detected in the oral cavity and also exists in IE (Hahn et al., 2005; Miao et al., 2024). The oral *Streptococcus* could promote the monocyte-to-DC differentiation, lowering the abundance of macrophages, which is not conducive to the clearance of pathogens (Hahn et al., 2005). In other oral microbiomes, the HACEK group pathogens, consisting of *Haemophilus parainfluenzae, Aggregatibacter* spp.*, Cardiobacterium* spp.*, Eikenella corrodens, and Kingella* spp., have been reported to cause IE, in which the *A. actinomycetemcomitans* exerts the strongest correlation (Revest et al., 2016; Nørskov-Lauritsen et al., 2019). *A. actinomycetemcomitans* endocarditis exhibits a longer duration and delayed diagnosis due to specific culture demands (Talapko et al., 2024). *A. actinomycetemcomitans* has also been reported to bind to acid-solubilized type I collagen through the extracellular matrix adhesin A (EmaA), which is highly expressed in heart valves (Del Giudice et al., 2021). Other pathogens, including *Enterococcus faecalis, Eikenella corrodens, Rothia dentocariosa, Lactobacillus gasseri, Lactobacillus rhamnosus*, and *Neisseria elongata*, have also been reported to cause IE in case reports (Broeren and Peel, 1984; Bígl et al., 1990; Haddow et al., 2003; Okui et al., 2015; Elikowski et al., 2017; Sweedan et al., 2023).

Oral hygiene is significantly associated with IE. Recent studies have shown that iron availability can substantially influence the composition of the oral microbiome, thereby affecting the development of IE. Salivary microbes were cultured in an iron-deficient environment *in vitro*, and it was found that hemolytic bacterial species, including *Streptococcus* spp.*, Gemella* spp., and *Granulicatella* spp., were significantly enriched (Wang et al., 2012). Another cross-sectional study found a distinct composition and lower diversity in patients with iron-deficiency anaemia (Xi et al., 2019). However, the mechanisms of how iron affects changes in bacterial composition remain to be investigated. Dental manipulations are thought to increase the risk of bacteremia, which in turn promotes IE. The risk of bacteremia with periodontal procedures, including subgingival irrigation, periodontal probing, scaling, and root planning, increases with the severity of periodontitis (Daly et al., 2001; Kinane et al., 2005; Forner et al., 2006a). Using a diode laser in conjunction with ultrasonic scaling has been found to reduce bacteremia owing to reduced tissue trauma and the antibacterial activity of the laser (Assaf et al., 2007). Despite the immediate potential risk of bacteremia, regular professional dental care is still advised for patients at risk of IE to reduce the source of bacterial seeding (Writing Committee Members et al., 2021). As for orthodontic procedures, the embedding of separators may cause bacteremia, while other orthodontic procedures have not been reported to increase bacteremia (Lucas et al., 2002). The incidence of bacteremia after tooth extraction rises with the increase in oral bacterial burden (Parahitiyawa et al., 2009). The use of PVP-iodine solution in subgingival scaling and the topical administration of vancomycin have been linked to a lower risk of bacteremia (Borthen Svinhufvud et al., 1988). However, the ACC/AHA guidelines recommend the prophylactic use of antibiotics only for individuals at the highest risk of infective endocarditis (Writing Committee Members et al., 2021). Therefore, the use of prophylactic antibiotics should be exercised with greater caution.

## Oral microbiome and heart failure

Heart failure (HF) is the terminal stage of various cardiovascular diseases, and the research concerning oral microbiomes and HF remains limited. Periodontal status and oral microbiome diversity are associated with incident HF (Molinsky et al., 2022; Yuzefpolskaya et al., 2023). Specifically, higher HF morbidity has been observed in patients with an increased level of *P. gingivalis* antibodies (Lai et al., 2025). Periodontitis caused by *P. gingivalis* infection accelerates the progression of HF with preserved ejection fraction in mice, which may be related to the systemic inflammation elevation and NO depletion (Daana et al., 2025). Reduced nitrate-reducing capacity has been observed in patients with HF with reduced ejection fraction (HFrEF), and modulation of the oral microbiome as well as oral inorganic nitrate supplementation may represent potential therapeutic strategies (Stahl et al., 2025). A Higher level of inflammation markers was observed in the plasma of HF patients, which can be reduced in those receiving oral hygiene procedures (Czerniuk et al., 2017; Dekker et al., 2017). Moreover, the elevated abundance of *F. prausnitzii* and butyrate in the gut was observed in HF patients, but the association between specific species of oral bacteria and HF remains to be explored (Zhang et al., 2025). Oral microbiome has been detected to translocate to the gut, and gut dysbiosis has been related to promoting chronic inflammation and the deterioration of gut endothelial barrier function (Kitai and Tang, 2018). On the other hand, impaired left ventricular function can result in intestinal edema and microcirculatory disturbances, leading to epithelial ischemia and disruption of the intestinal barrier, which may further exacerbate cardiac dysfunction and contribute to the progression of HF (Rogler and Rosano, 2014).

## Discussion

The association between the oral microbiome and cardiovascular diseases is a specific reflection of human health. Accumulating evidence suggests that the occurrence and development of CVD are often accompanied by changes in the oral microbial community and their ectopic colonization. Although the association between dysbiosis of the oral microbiome and CVD is clear, the underlying mechanisms still remain obscure. The elucidation of complex mechanisms linking oral bacteria and CVD, including microbial virulence factors, endothelial dysfunction, platelet aggregation, immune activation, bacteremia, and systemic inflammation, gradually fills the ongoing gap in our understanding of CVD. Therefore, a systematic review of the association between the oral microbiome and CVD will provide new perspectives for key cutting-edge research and contribute to the prevention and management of cardiovascular health.

The epidemiological correlations between oral microbiota and CVD are gradually established. Most observational studies have demonstrated a significant relationship between CVD morbidity and mortality and the composition and diversity of the oral microbiome in the saliva (Byun et al., 2020; Ding et al., 2024; Adam et al., 2025; Akhi et al., 2025). However, research focusing on periodontal diseases and CVD has mainly relied on periodontal probing, and future studies should collect dental plaque from patients to conduct accurate studies. Furthermore, most studies focus on the whole population, and a few studies have begun to explore specific subgroups of the population according to age, gender, and other factors. Accurate research on high-risk populations may lead to more targeted conclusions. Interventional studies showed controversial conclusions of periodontal treatment and cardiovascular status improvement, which is probably due to small sample size and different follow-up durations (Forner et al., 2006b; Waghmare et al., 2013; Abdullah et al., 2024). Thus, intervention studies with larger sample sizes and multi-point monitoring spanning longer follow-up periods are urgently needed.

A considerable number of studies have explored the mechanisms underlying the association between the oral microbiome and CVD. Microbiota in the oral cavity have been proven to induce vascular endothelial dysfunction, which subsequently aggravates the progression of CVD through promoting bacteria entering the bloodstream, as confirmed by the detection of oral microbial DNA in atherosclerotic plaques (Waghmare et al., 2013; Li et al., 2022; Miyauchi et al., 2025). Meanwhile, animal studies showed that the accumulation of oral microbiota in lymph nodes could induce inflammation, which subsequently promotes the progression of CVD (Chen, 2025). As a chronic inflammatory disease, periodontitis has been found to cause elevated leukocyte numbers, IL-6, and hs-CRP, and the lower level of systemic inflammation may promote CVD (Schulze-Späte et al., 2024). Furthermore, animal studies showed the activation of monocytes/macrophages in experimental periodontitis, which adhered to vascular endothelial cells, causing endothelial dysfunction and the initiation of atherosclerosis (Miyajima et al., 2014). Periodontitis has also been reported to induce inflammation-induced trained myelopoiesis, causing long-lasting epigenetic rewiring was identified in HSPC (Li et al., 2023). However, limited research has been conducted on trained immunity and its association with adaptive immunity in the context of oral microbiome and cardiovascular disease. Therefore, more studies are encouraged to prioritize these areas to advance understanding and inform potential therapeutic strategies. Large-scale studies with various disease stages are essential to trace the dynamic changes of the oral microbiome with the progression of CVD and develop predictive tools for CVD prognosis based on the oral microbiome. Concerning treatment, greater emphasis could focus on eliminating specific pathogens beyond conventional periodontal therapy, such as employing bacteriophages to eradicate pathogenic bacteria or developing vaccines for preventive purposes.

Overall, this article reviewed the current clinical research on the link between oral microbiome and CVD and discussed the underlying pathophysiological mechanisms. However, the limitation of this review was that only common CVD were investigated, and relatively rare CVD such as abdominal aortic aneurysm or atrial fibrillation were not explored. Correlation mechanisms such as neutrophil extracellular traps have not been elaborated in detail. It will be essential to conduct more comprehensive and rigorous studies that include investigations into the abundance of the oral microbiome across populations with strict control of baseline conditions and explore the underlying mechanisms behind the correlations.
